# Evaluation of *Salipiger thiooxidans* and *Exiguobacterium aestuarii* from the Saemangeum Reservoir as Potential Probiotics for Pacific White Shrimp (*Litopenaeus vannamei*)

**DOI:** 10.3390/microorganisms10061113

**Published:** 2022-05-27

**Authors:** Soohwan Kim, Hyuncheol Jeon, Sungchul Charles Bai, Jun-Wook Hur, Hyon-Sob Han

**Affiliations:** 1 Faculty of Marine Applied Biosciences, Kunsan National University, Gunsan 54150, Korea; soohwan@kunsan.ac.kr; 2 Department of Marine Bio-Material & Aquaculture, Pukyong National University, Busan 48513, Korea; conjp@naver.com; 3 Feeds & Foods Nutrition Research Center, Pukyong National University, Busan 48547, Korea; scbai@pknu.ac.kr; 4 FAO World Fisheries University Pilot Program, Busan 48547, Korea

**Keywords:** *Salipiger thiooxidans*, *Exiguobacterium aestuarii*, *Litopenaeus vannamei*, Saemangeum Reservoir, probiotics, water additives

## Abstract

In this study, two bacterial species, *Salipiger thiooxidans* and *Exiguobacterium aestuarii*, were extracted and screened from the Saemangeum Reservoir. This study examined these species’ suitability as a probiotic by confirming the effects of *S. thiooxidans* and *E. aestuarii* added to rearing water for *L. vannamei*. Three experimental groups were evaluated for 6 weeks: (1) a control group reared in natural (i.e., untreated) water (CON), (2) an experimental group in which *S. thiooxidans* was added to natural water (SMG-A), and (3) natural water inoculated with *E. aestuarii* (SMG-B). The SMG-B group inoculated with *E. aestuarii* showed significantly higher final body weight, weight gain, specific growth rates, and feed efficiency than the control group. The SMG-B group inoculated with *E. aestuarii* exhibited significantly higher levels of serum lysozyme, and ACP and ALP activity than the control and SMG-A groups. The SMG-A and SMG-B groups inoculated with probiotics showed significantly lower total ammonia nitrogen and nitrite than the control group. Our findings suggest that *S. thiooxidans* and *E. aestuarii* extracted from the Saemangeum Reservoir can improve the water quality of aquaculture water, and, in particular, *E. aestuarii* is a potential probiotic for *L. vannamei*.

## 1. Introduction

Aquaculture is one of the most promising sources of animal protein [1]. However, in recent years, due to indiscriminate intensive aquaculture, water quality deterioration and diseases (viruses and bacteria) have become a serious threat to the aquaculture industry [2]. Although antibiotics and other therapeutic compounds have traditionally been used to treat diseases, these methods are no longer recommended, as they have been linked to negative effects such as antibiotic resistance, antibiotic residues, and environmental pollution [3]. These limitations thus highlight the urgent need to develop alternatives to antibiotics.

An alternative to antibiotic treatment is to apply probiotics [4]. Probiotics are defined as “living microorganisms that, when ingested in adequate amounts, confer a health benefit on the host” [5]. In the past few decades, probiotics have been actively studied for a variety of purposes, including pharmaceutical products, functional foods, wastewater treatment, livestock, and aquaculture [6,7,8,9,10]. Probiotics are being actively studied as an alternative to antibiotics due to their advantages of not generating residues or drug resistance in the body of animals [11]. Probiotics are promising and environmentally friendly alternatives to antibiotics in preventing aquaculture diseases [12]. In the field of aquaculture, probiotics are supplied to farmed fish by adding them to rearing water and feed, and have the advantages of improving the growth and feed efficiency of farmed fish, inhibiting the proliferation of pathogenic microorganisms, and improving rearing water quality [13,14,15].

*Litopenaeus vannamei* is an aquaculture species favored by consumers internationally; its aquaculture production reached 4.97 million tonnes in 2018, and its production has been steadily increasing [16]. Probiotics are becoming a more popular alternative to prevent disease, especially in the aquaculture of *L. vannamei*, which is relatively susceptible to disease compared to fish [17]. In addition to inhibiting the growth of pathogenic microbes, the live microbes of microbial probiotics enhance the growth performance, gut digestive enzymes, feed efficiency and absorption, and immune responses of shrimp [18]. A typical example of adding useful microorganisms such as probiotics to water is the aquaculture system using the biofloc technology [19]. Since its inception at the French Research Institute for Exploitation of the Sea (IFREMER) in the 1970s, research regarding biofloc technology has increased from fewer than 10 publications in 2009 to more than 100 publications in 2018, and research has been conducted mainly in Brazil, China, the United States, Mexico, India, and Thailand [20]. As reported by the scientific community and academia, despite the advances and benefits of BFT, the potential and room for commercial expansion still exist; for example, it is estimated that only about 10% and 20% of the shrimp production in South Korea and Indonesia, respectively, use biofloc technology [20]. One of the reasons for such low levels is that the implementation and reproducibility of biofloc technology are complicated. In most biofloc studies using useful microorganisms such as probiotics in water for shrimp, the species constituting the microbial community are not clearly specified in the reports [21,22,23,24,25,26,27,28,29,30,31,32]. To date, about 20 bacterial genera have been reported to have probiotic effects in shrimp, but most studies have focused only on lactic acid bacteria such as *Bacillus* spp. and *Lactobacillus* spp. [33]. There are various problems related with the probiotic products containing *Bacillus* spp. and *Lactobacillus* spp. available in South Korea, Thailand, and other countries, such as their high cost and ineffectiveness [34]. A recent study of bacterial populations in shrimp biofloc farms using probiotics showed that, in addition to *Bacillus* species, *Exiguobacterium* sp. and *Salipiger* sp. were dominant in stock water, and these bacteria provided a favorable environment for shrimp farming by removing organic compounds from aquaculture waste [35,36]. Despite the advantages that can be utilized as probiotics, research on these microorganisms is lacking compared to that on *Bacillus* and *Lactobacillus*, which are relatively active in research.

In this study, two bacterial species, *Salipiger thiooxidans* and *Exiguobacterium aestuarii*, extracted and screened (fast-growing) from the Saemangeum Reservoir, the same environment as in the previous study [37], were selected and tested. This study aimed to assess the suitability of using *S. thiooxidans* or *E. aestuarii* in the rearing water of *L. vannamei* as a probiotic for this shrimp, by evaluating the growth performance, hemolymph parameters, and water quality.

## 2. Materials and Methods

### 2.1. Ethics Statement

This study followed the guidelines of the Institutional Animal Care and Use Committee regulations issued by the Kunsan National University, Gunsan, Republic of Korea. Every effort was taken to minimize shrimp suffering.

### 2.2. Extraction of Microorganisms from Saemangeum Reservoir

**Figure 1:**
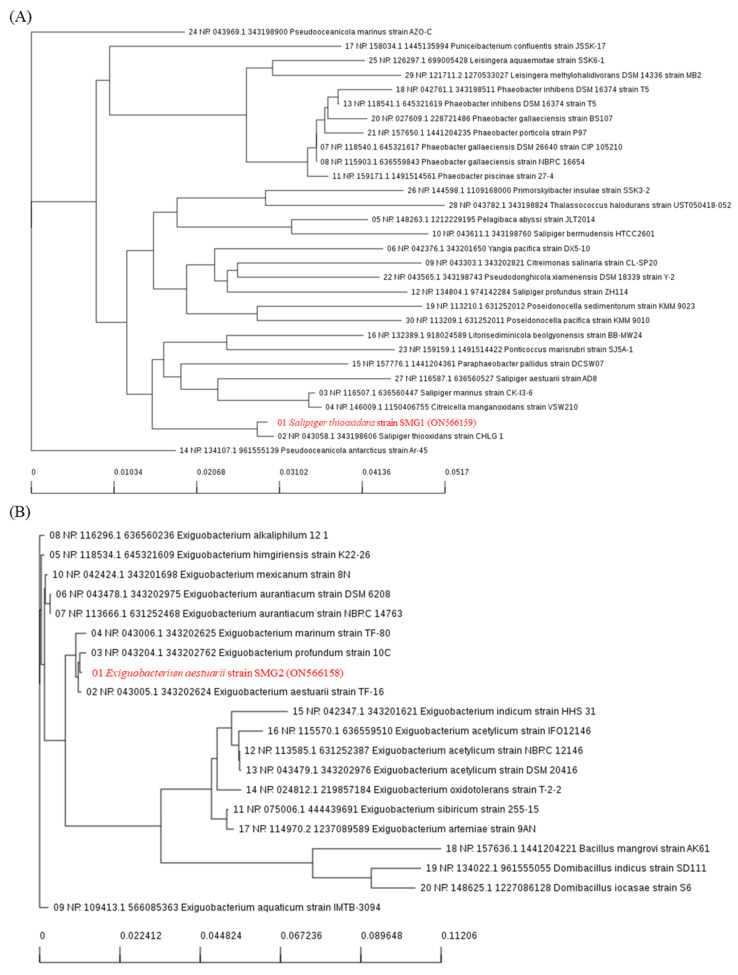
Phylogenetic tree derived from neighbor-joining analysis of partial 16S rRNA gene sequences of effective microorganisms and their closely related species: (**A**) *Salipiger thiooxidans* strain SMG1, (**B**) *Exiguobacterium aestuarii* strain SMG2.

Bacteria samples were collected from the Saemangeum Reservoir (35°52′07.3″ N, 126°30′29.8″ E) located in Jeonbuk Province, Republic of Korea. Both bacterial strains were inoculated as a spot (diameter 2 mm) on the surface of a LB agar plate spread with cell suspension of a given indicator strain. Cells were incubated 48 h at 25 °C, and bacterial species were identified using 16S rDNA sequencing. The microorganism genomes were sequenced using the BigDye Terminator v3.1 cycle sequencing kit (Applied Biosystems, Hampton, NY, USA) according to the manufacturer’s instructions after PCR amplification using the 27F-CM (5′-AGA GTT TGA TCM TGG CTC AG-3′) and 1492R (5′-TAC GGY TAC CTT GTT A-C GAC TT-3′) primer pairs [37,38]. A BLAST similarity search of the 16S rRNA was conducted of the National Centre for Biotechnology Information (NCBI) database using BLASTN. Analysis of the phylogenetic tree construction was generated using the neighbor-joining statistical method. The sequences were submitted to GenBank, where they have been assigned the *S. thiooxidans* strain SMG1 (GenBank Accession No. ON566159) and the *E. aestuarii* strain SMG2 (GenBank Accession No. ON566158) (Figure 1).

### 2.3. Shrimp and Experimental Condition

The experiment was carried out in an indoor shrimp farming facility at Kunsan National University (Gunsan, Korea). *L. vannamei* juveniles were purchased from a shrimp farm (Taean, Korea). The shrimps were allowed to acclimatize to the experimental conditions and facility for 15 d, during which they were fed with shrimp feed containing 38.5% crude protein and 5.4% crude lipid. After the acclimatization period, similarly sized shrimp (mean ± standard deviation, 0.40 g ± 0.01) were randomly stocked in 50 L acrylic tanks (9 tanks with 40 shrimps per tank). Three experimental groups were evaluated in this experiment: (1) a control group reared in natural (i.e., untreated) water (CON), (2) an experimental group in which *Salipiger thiooxidans* was added to natural water (SMG-A), and (3) natural water inoculated with *Exiguobacterium aestuarii* (SMG-B). The two experimental groups were raised in water inoculated weekly with probiotics (10^6^ CFU/mL) containing *S. thiooxidans* (SMG-A) or *E. aestuarii* (SMG-B). Both bacterial strains were cultured using LB medium (Luria-Bertani, LB Broth, Miller, BD DifcoTM, Sparks, MD, USA), and both species were cultured at 25–26 °C, pH 7 ± 0.5, and 15 psu (salinity). The shrimp were fed four times a day (at 8:00, 12:00, 16:00, and 20:00), and the feed ratios were adjusted to 8–16% of the body-weight of the shrimps for 6 weeks. All the experiments were conducted using a static system and the rearing water was not exchanged (zero-exchange water). The average water temperature during the rearing experiments was maintained at 29.5–30.0 °C. Appropriate dissolved oxygen levels were maintained by installing air stones in all experimental water tanks, and a 12:12 (light:dark) photoperiod was maintained using a fluorescent lamp.

### 2.4. Growth Performance and Sample Collection

After undergoing weight measurements, the shrimps were fasted for 20 h, and growth was measured in terms of total shrimp weight. After the final weighing, five shrimps from each tank were randomly selected and anesthetized in ice water for innate immunity analysis. The hemolymph of the shrimp was collected using a syringe treated with Alsever’s solution (Sigma-Aldrich, St. Louis, MO, USA), and the samples were allowed to clot at room temperature for 30 min. The serum was then separated by centrifugation (4 °C, 5000× *g*, 10 min). Microorganisms (100 g) were freeze-dried and used for component analysis.

### 2.5. Proximate Composition Analysis

Proximate composition analysis was performed using the standard AOAC methods [39]. The samples were first freeze-dried for 48 h before the proximate analyses. Moisture level was determined by drying the samples in an oven at 105 °C, and ash content was quantified by combustion at 550 °C. Crude protein content was quantified via the Kjeldahl method, and crude lipid levels were determined via Soxhlet extraction by using a Tecator 1046 Soxhlet system (Tecator AB, Munkedal, Sweden) [40].

### 2.6. Amino Acid Analysis

The two bacterial strains (probiotics) and carcasses of shrimp were freeze-dried for amino acid analysis [37]. Each sample (0.02 g) was hydrolyzed with 15 mL of 6 M HCl at 110 °C for 24 h. The samples were hydrolyzed in distilled water in a 50 mL flask, then evaporated and recovered in sodium citrate buffer (pH 2.2). After filtration (0.2 µm), the samples were analyzed using ninhydrin at 570 nm and 440 nm in an S433 amino acid analyzer (Sykam, Gilching, Germany). For methionine and cystine hydrolysis, performic acid was used instead of 6 M HCl.

### 2.7. Innate Immunity Analysis

The parameters of acid phosphatase (ACP), alkaline phosphatase (ALP), and lysozyme activity in hemolymph were determined by a GENESYS™ 10S UV-Vis Spectrophotometer (Thermo Fisher Scientific Inc., Vantaa, Finland). The activity of TRACP and ALP was analyzed using the TRACP & ALP Assay Kit (Takara Korea, Seoul, Korea). Lysozyme activity was analyzed using the Lysozyme Activity Assay Kit (BioVision Inc., Milpitas, CA, USA). The parameters of all measured indexes were adjusted according to the instructions.

### 2.8. Water Quality Analysis

The water of all experimental tanks was collected every three days and used for water quality analysis. The rearing water of tanks was passed through a 0.2 μm syringe filter, and the total ammonia nitrogen (TAN, NH_4_^+^-N and NH_3_-N), nitrite (NO_2_^−^), nitrate (NO_3_^−^), and phosphate (PO_4_^3−^) concentrations of water samples were quantified by Water-quality Assay Kit (Humas Co Ltd., Daejeon, Korea). All samples were measured according to the manufacturer’s instructions. Dissolved oxygen (DO) and pH were measured using a YSI MultiLab 4010-3 water quality analyzer (Yellow Springs, OH, USA).

### 2.9. Statistical Analysis

All data were analyzed using one-way analysis of variance (ANOVA), followed by the Tukey HSD test in SPSS Statistics for Windows, version 26.0 (IBM Corp., Armonk, NY, USA). Statistical significance was determined at *p* < 0.05. All the data are reported as mean ± SD. Percentage data were arcsine transformed prior to analysis.

## 3. Results

### 3.1. Growth Performance and Whole-Body Composition

Table 1 summarizes the growth, feed efficiency (FE), and survival rate of the CON, SMG-A, and SMG-B groups after the 6-week culture period. The SMG-B group inoculated with *E. aestuarii* showed significantly higher final body weight (FBW) and weight gain (WG) than the control and SMG-A groups (*p* < 0.05). The SMG-B group showed significantly higher FE than the control group (*p* < 0.05). There was no significant difference in the survival rate among all the groups.

Table 2 and Table 3 show the results of the whole-body proximate and amino acid composition of shrimp after the 6-week culture period. The crude protein level of the shrimp in the SMG-B group was significantly higher than that of the control and SMG-A groups. There was no significant difference in the levels of the crude lipid and crude ash among the all groups. There was no significant difference in the proximate compositions between the two microorganisms *S. thiooxidans* and *E. aestuarii* in the experiment. The levels of arginine, histidine, glutamic acid, and glycine in the whole body of the shrimp in the SMG-A or SMG-B groups were significantly higher than those in the control group. Leucine and phenylalanine levels were significantly higher in the shrimp whole body of the SMG-B group than in the control group. In addition, the SMG-B group showed the highest lysine and glycine levels among all the groups. However, the whole body of the shrimp in the control group showed a significantly higher proline level than that in any other group.

### 3.2. Innate Immune Response

Table 4 shows the results of the assessment of the innate immunity in the hemolymph of Pacific white shrimp after the 6-week culture period. The shrimp in the SMG-B group, inoculated with *E. aestuarii*, showed significantly higher serum lysozyme levels than the control and SMG-A groups. Additionally, serum ACP and ALP activity was significantly higher in the SMG-B group than in the control and SMG-A groups.

### 3.3. Water Quality

**Figure 2:**
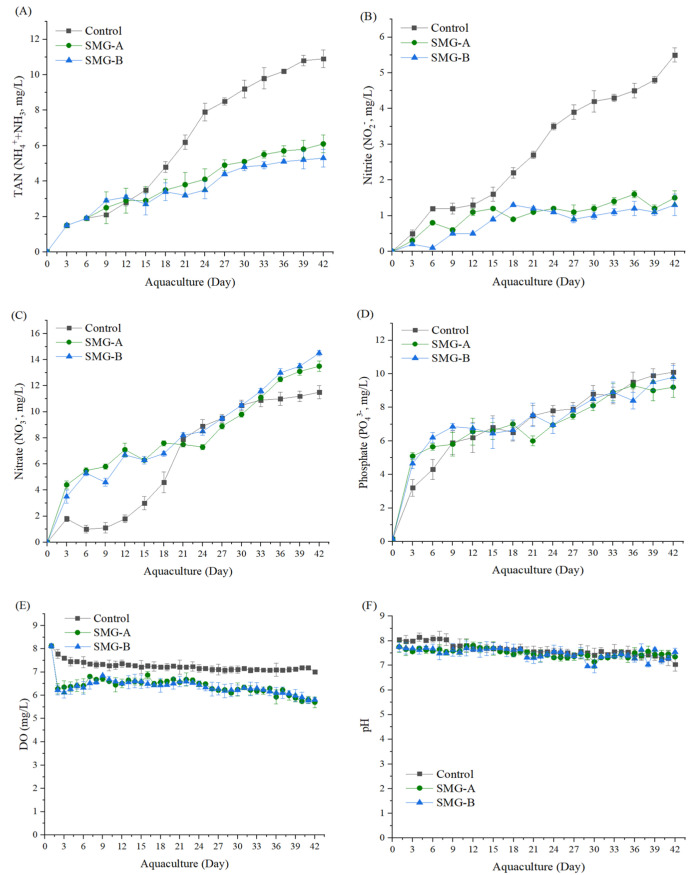
Change patterns of (**A**) TAN (NH_4_^+^ and NH_3_), (**B**) nitrite (NO_2_^−^), (**C**) nitrate (NO_3_^−^), (**D**) Phosphate (PO_4_^3−^), (**E**) DO, and (**F**) pH concentrations experimental tanks during the 6-week study period. The data points represent the treatment means and the error bars represent the standard error of the mean (SEM).

Figure 2 shows the change in the water quality of the rearing water in the Pacific white shrimp experimental tank during the 6-week culture period. There was no significant difference in phosphate, DO, and pH levels in rearing water in all groups. The SMG-A and SMG-B groups showed significantly lower TAN and nitrite levels than the control group. The nitrate level in the control group was significantly lower than in the SMG-A and SMG-B groups. The phosphate levels in all the groups similarly increased during the culture period.

## 4. Discussion

Research utilizing probiotics in water, such as the biofloc system, mostly relies on local water resources (unspecified microbial communities such as heterotrophs, microalgae, protozoa, and yeast, presumably present in the influent). Since the microbial species constituting the biofloc technology cannot be clearly identified, it is practically difficult to utilize probiotics in shrimp farms in other regions. Microorganisms in natural seawater may have a positive effect on shrimp or may coexist with pathogenic microorganisms, such as *Vibrio* species, that also proliferate using carbon sources [42]. If the probiotic bacteria are relatively dominant, the growth of pathogenic microorganisms may be inhibited; however, the opposite case may cause mass death of shrimp, so caution is required. Therefore, many shrimp farms that cannot find a water resource with an effective microbial community in a nearby watershed purchase expensive probiotic products and use them for aquaculture. However, there are no strict regulations for probiotic products for aquaculture in some regions, and there are concerns about labeling reliability because additional species other than those specified by manufacturers in commercial probiotic products are being detected [33,43]. Commercial probiotics often detect more than one microbial species, in addition to multiple additional microorganisms other than those specified in the product, making it difficult to characterize the mechanism for the probiotic effect, or to attribute the effect to a single species [33]. Therefore, for sustainable aquaculture, accurate information disclosure and research on microbial strains used in probiotics are required, and it is necessary to discover and develop probiotics optimized for each aquaculture species.

In numerous studies, probiotic bacteria have been reported to provide nutrients and increase digestive enzyme activity, thereby improving host growth and feed efficiency [44]. Probiotics, such as *Bacillus* spp. and *Lactobacillus* spp., have been reported to improve shrimp growth and reduce feed costs by producing amylases, proteases, lipases, and cellulases in the intestine to improve the digestion and absorption of proteins by the shrimp [17,45,46]. The study presented here showed that the administration of *E. aestuarii* to the rearing water can significantly improve the growth and FE of *L. vannamei*. *Exiguobacterium* sp. has been shown to produce protease over a wide temperature range and has been reported to produce several effective enzymes, including proteolytic enzymes [47]. In this study, the levels of crude protein, essential amino acids (lysine, leucine, phenylalanine, histidine, and arginine) and non-essential amino acids (glutamic acid and glycine) of the shrimp reared with *E. aestuarii* were significantly higher than the control levels. Essential amino acids are nutrients that must be supplied as factors that affect the growth of shrimp [48,49,50]. Probiotics provide essential amino acids to the host by breaking down complex nutrients in the intestine for growth, so intake of probiotics improves intestinal microbial balance to increase nutrition absorption, increase feed efficiency, and improve the growth rate of shrimp [51]. It is considered that *E. aestuarii* administrated in the water was ingested by *L. vannamei* and produced proteolytic enzymes in the body of the shrimp to improve the absorption of proteins and essential amino acids by the shrimp. Therefore, the significant increases in the growth performance of the shrimp in the SMG-B group were likely due to the nutritional improvement caused by the administration of *E. aestuarii*. In this study, various amino acid levels were increased according to *E. aestuarii* administration, except for the proline level, which was significantly higher in the control group (not inoculated with any probiotic) than in the other groups. After adding proline to the feed for 8 weeks, no significant change in the growth of the shrimp was detected [52]. According to a previous study, the blood proline level of infants fed formula containing the probiotic *Bifidobacterium lactis* was significantly lower than that of infants fed formula without probiotics [53]. Therefore, it is presumed that probiotics can affect the reduction of proline in the body, but further research is needed on the relationship between probiotics and proline in *L. vannamei*. This is the first study in which *E. aestuarii* alone was inoculated into rearing water and tested on *L. vannamei*, and *E. aestuarii* was found to improve the growth performance, FE, and nutritional composition of *L. vannamei*.

The results of this study showed that the serum lysozyme level, and ACP and ALP activity, of *E. aestuarii* were significantly increased upon inoculation of the rearing water with either probiotic. Lysozyme catalyzes the hydrolysis of the β-(1,4)-glycosidic linkage of n-acetyl-glucosamine and n-acetyl-muramic acid, and thereby protects the host from bacterial infections [54]. Lysozyme activity is one of the most important immune factors in shrimp, and lysozyme is an antibacterial enzyme that acts on the cell wall of pathogens [55]. Similar to our study results, when *L. vannamei* was inoculated with *B. subtilis* and bred for 2 weeks, the lysozyme gene expression in *L. vannamei* was significantly increased [56]. In addition, when *Pediococcus pentasaceus* was added to the feed of *L. vannamei* for 8 weeks, the lysozyme activity of *L. vannamei* was significantly increased [57]. Phosphatase enzymes can hydrolyze organophosphate esters, and ACP and ALP are composed of several phosphomonoesterases that are very important for the crustacean immune system [58,59]. ACP is a lysosomal enzyme involved in killing and digesting invading organisms during the immune response, and ALP is a multifunctional enzyme that hydrolyzes various types of phosphomonoester substrates and acts as a transphosphorylase at alkaline pH [60,61]. Both ACP and ALP have been reported to correlate with the immune capacity of aquatic animals in immune defense mechanisms [59,62]. Similar to the results of this study, the rearing water of *Macrobrachium nipponense* was inoculated with *B. velezensis* CPA1-1. After 20 d, ACP and AKP activities were found to have significantly increased, and resistance to *Vibrio* infection was improved [63]. In addition, as a result of feeding *L. vannamei* with *Enterobacter hominis* and *Lactobacillus* sp. for 4 weeks, the activity of immune-related enzymes including ACP and AKP tended to increase [9]. These findings showed that probiotics enhance the immunity of shrimp by inducing the host’s immune enzyme levels [64]. Therefore, it is considered that *E. aestuarii* can increase the activity of immune-related enzymes and improve the non-specific immunity of *L. vannamei*.

In this study, the SMG-A and SMG-B groups, which were inoculated with the probiotics, had significantly lower TAN and nitrite levels than the control group. As the feed is supplied to the shrimp, the concentration of toxic ions, such as ammonia and nitrite, which increases due to the accumulation of feed residues and excrement in the rearing water, may increase, causing shrimp disease, and high mortality may occur [65]. Therefore, water quality management is an important factor in the success of shrimp aquaculture. Multiple studies have demonstrated that probiotics have great potential for reducing toxic ion concentrations in rearing water [65,66,67]. Similar to the results of this study, *B. subtilis* CM3.1 was inoculated into the rearing water of *L. vannamei*, and the growth parameters of the shrimp were observed for 120 h. TAN and nitrite levels were found to be significantly reduced [68]. In a similar study, the rearing water of the Pacific white shrimp was inoculated with *B. amyloliquefaciens*, and the growth parameters of the shrimp were observed for 169 d. Consequently, it was found that this bacterium can significantly improve water quality [69]. In a previous study, it was reported that *Salipiger* sp. can remove organic compounds, such as nitrite, from wastewater, and *E. aestuarii* has recently been shown to effectively eliminate such compounds from aquaculture wastewater through extracellular enzymatic activity [36,70]. In this study, it was confirmed that the water quality in the rearing water was improved due to the denitrification action by the two microorganisms. However, research on water quality improvement by these two microorganisms is insufficient, so it is considered that supplementary studies related to TSS and alkalinity are needed in the future.

## 5. Conclusions

Both bacterial strains were shown to improve the water quality in the aquaculture of *L. vannamei*. In particular, *E. aestuarii* can improve the growth, FE, nutritional content, and innate immunity of *L. vannamei*, so it can be recommended as a probiotic for *L. vannamei*. The synergistic effect of mixing the two bacterial strains, and mixing them with other proven probiotics, needs to be studied. Our findings suggest that *S. thiooxidans* and *E. aestuarii* from the Saemangeum Reservoir can improve the quality of aquaculture water, and, in particular, *E. aestuarii* is a potential probiotic for *L. vannamei*.

## Figures and Tables

**Table 1:** Growth performance of the Pacific white shrimp in the control, SMG-A, and SMG-B groups throughout the 6-week culture period ^1^.

	Control	SMG-A	SMG-B
Initial body weight (g)	0.40 ± 0.01	0.40 ± 0.01	0.40 ± 0.01
Final body weight (g)	4.67 ± 0.16 ^b^	4.88 ± 0.09 ^b^	5.48 ± 0.11 ^a^
WG (%) ^2^	1068.38 ± 41.19 ^b^	1191.01 ± 22.54 ^b^	1268.92 ± 26.59 ^a^
FE (%) ^3^	90.05 ± 7.43 ^b^	94.71 ± 2.72 ^ab^	108.26 ± 6.53 ^a^
Survival (%) ^4^	90.83 ± 3.82	93.33 ± 1.44	91.67 ± 6.29

^1^ Values are means from triplicate groups of shrimps where the values in each row with different superscripts are significantly different (*p* < 0.05). ^2^ Weight gain (WG, %) = (final weight − initial weight) × 100/initial weight. ^3^ Feed efficiency rates (FE, %) = (wet weight gain/dry feed intake) × 100. ^4^ Survival rate (%) = (initial number of shrimp − dead shrimp) × 100/initial number of shrimps.

**Table 2:** Whole-body proximate compositions of the Pacific white shrimp cultured in the control, SMG-A, and SMG-B groups after 6 weeks of culture ^1^.

(%)	Pacific White Shrimp ^2^			Microorganisms ^3^	
	Control	SMG-A	SMG-B	*S. thiooxidans*	*E. aestuarii*
Moisture	71.95 ± 0.25	71.46 ± 0.65	71.28 ± 0.59	0.42 ± 0.02	0.54 ± 0.01
Crude protein	19.87 ± 0.23 ^b^	19.99 ± 0.35 ^b^	23.11 ± 0.41 ^a^	71.65 ± 0.62	72.23 ± 0.84
Crude lipid	1.48 ± 0.11	1.53 ± 0.08	1.65 ± 0.10	0.61 ± 0.02	0.66 ± 0.05
Crude ash	3.65 ± 0.27	3.88 ± 0.24	3.75 ± 0.31	8.40 ± 0.18	8.45 ± 0.09

^1^ Values are means from triplicate groups of shrimps where the values in each row with different superscripts are significantly different (*p* < 0.05). ^2^ Wet weight basis. ^3^ Dry matter basis.

**Table 3:** Whole-body amino acid compositions (mg 100 mg^−1^) of the Pacific white shrimp cultured in the control, SMG-A, and SMG-B groups after 6 weeks of culture ^1^.

	Pacific White Shrimp ^2^			Microorganisms ^2^	
	Control	SMG-A	SMG-B	*S. thiooxidans*	*E. aestuarii*
**Essential amino acids (EAA) ^3^**					
Arginine	4.60 ± 0.08 ^b^	5.10 ± 0.10 ^a^	5.37 ± 0.33 ^a^	2.25 ± 0.05	2.71 ± 0.13
Threonine	2.88 ± 0.05	3.21 ± 0.03	2.85 ± 0.24	2.02 ± 0.02	2.01 ± 0.05
Valine	2.88 ± 0.02	3.18 ± 0.02	3.28 ± 0.23	2.98 ± 0.12	3.42 ± 0.08
Isoleucine	2.52 ± 0.07	2.72 ± 0.03	2.86 ± 0.29	2.24 ± 0.03	2.75 ± 0.06
Leucine	4.04 ± 0.12 ^b^	4.40 ± 0.05 ^ab^	4.67 ± 0.34 ^a^	3.21 ± 0.02	3.55 ± 0.08
Methionine	1.43 ± 0.02	1.46 ± 0.04	1.45 ± 0.02	0.49 ± 0.02	0.68 ± 0.03
Lysine	4.27 ± 0.01 ^b^	4.34 ± 0.08 ^b^	5.02 ± 0.37 ^a^	3.63 ± 0.05	4.12 ± 0.04
Phenylalanine	2.59 ± 0.12 ^b^	2.79 ± 0.04 ^ab^	3.00 ± 0.23 ^a^	2.09 ± 0.11	2.29 ± 0.14
Histidine	2.77 ± 0.04 ^b^	3.18 ± 0.06 ^a^	3.54 ± 0.12 ^a^	1.98 ± 0.06	2.08 ± 0.11
**Non-essential amino acids (NEAA) ^3^**					
Serine	2.35 ± 0.01	2.67 ± 0.04	2.63 ± 0.07	1.65 ± 0.04	1.58 ± 0.09
Glutamic acid	9.46 ± 0.03 ^b^	10.55 ± 0.16 ^a^	10.53 ± 0.06 ^a^	6.53 ± 0.22	7.52 ± 0.18
Proline	10.42 ± 0.01 ^a^	6.36 ± 0.15 ^b^	4.98 ± 0.79 ^c^	1.88 ± 0.05	1.00 ± 0.04
Glycine	4.40 ± 0.01 ^c^	4.89 ± 0.07 ^b^	5.38 ± 0.16 ^a^	2.45 ± 0.09	2.55 ± 0.14
Alanine	2.33 ± 0.01	2.53 ± 0.13	2.56 ± 0.09	4.13 ± 0.46	4.26 ± 0.22
Tyrosine	1.94 ± 0.18	1.97 ± 0.06	2.16 ± 0.17	1.17 ± 0.13	1.50 ± 0.14
Aspartic acid	7.87 ± 0.07	7.90 ± 0.03	7.89 ± 0.51	4.17 ± 0.22	3.25 ± 0.07
Cysteine	1.51 ± 0.02	1.43 ± 0.03	1.56 ± 0.01	0.53 ± 0.08	0.50 ± 0.05

^1^ Values are mean of triplicate groups and presented as mean ± SD. Values in the same row having different superscript letters are significantly different (*p* < 0.05). ^2^ Dry matter basis. ^3^ The concept of essential amino acids of *Litopenaeus vannamei* refers to the National Research Council (2011) [41].

**Table 4:** Non-specific immune parameters of the Pacific white shrimp in the control, SMG-A, and SMG-B groups after 6 weeks of culture.

	Control	SMG-A	SMG-B
Lysozyme ^1^	0.128 ± 0.006 ^b^	0.141 ± 0.017 ^b^	0.206 ± 0.011 ^a^
ACP ^2^	11.95 ± 0.23 ^b^	12.38 ± 0.48 ^b^	20.35 ± 0.30 ^a^
ALP ^3^	2.08 ± 0.08 ^b^	2.19 ± 0.13 ^b^	4.32 ± 0.58 ^a^

Values are means of triplicate groups and presented as mean ± S.D. Values with different superscripts in the same column are significantly different (*p* < 0.05). ^1^ Lysozyme activity (U mL^−1^). ^2^ Acid phosphatase (U mL^−1^). ^3^ Alkaline phosphatase (U mL^−1^).

## Data Availability

All data is reported in this article.
